# Coastline changes: A reconsideration of the prevalence of recession on sandy shorelines – CORRIGENDUM

**DOI:** 10.1017/cft.2026.10031

**Published:** 2026-05-15

**Authors:** Colin D. Woodroffe, Niki Evelpidou, Irene Delgado-Fernandez, David Green, Dhritiraj Sengupta, Anna Karkani, Paolo Ciavola

The author’s name was incorrectly spelled in the article as originally published (Dhriti Sengupta).

This has been corrected to Dhritiraj Sengupta in the HTML and PDF versions of the article.

We publish this notice for the scholarly record.
